# TUGDA: task uncertainty guided domain adaptation for robust generalization of cancer drug response prediction from *in vitro* to *in vivo* settings

**DOI:** 10.1093/bioinformatics/btab299

**Published:** 2021-07-12

**Authors:** Rafael Peres da Silva, Chayaporn Suphavilai, Niranjan Nagarajan

**Affiliations:** School of Computing, National University of Singapore, 117417 Singapore, Singapore; Genome Institute of Singapore, A*STAR, 138672 Singapore, Singapore; Genome Institute of Singapore, A*STAR, 138672 Singapore, Singapore; School of Computing, National University of Singapore, 117417 Singapore, Singapore; Genome Institute of Singapore, A*STAR, 138672 Singapore, Singapore; Yong Loo Lin School of Medicine, National University of Singapore, 119228 Singapore, Singapore

## Abstract

**Motivation:**

Large-scale cancer omics studies have highlighted the diversity of patient molecular profiles and the importance of leveraging this information to deliver the right drug to the right patient at the right time. Key challenges in learning predictive models for this include the high-dimensionality of omics data and heterogeneity in biological and clinical factors affecting patient response. The use of multi-task learning techniques has been widely explored to address dataset limitations for *in vitro* drug response models, while domain adaptation (DA) has been employed to extend them to predict *in vivo* response. In both of these transfer learning settings, noisy data for some tasks (or domains) can substantially reduce the performance for others compared to single-task (domain) learners, i.e. lead to negative transfer (NT).

**Results:**

We describe a novel multi-task unsupervised DA method (TUGDA) that addresses these limitations in a unified framework by quantifying uncertainty in predictors and weighting their influence on shared feature representations. TUGDA’s ability to rely more on predictors with low-uncertainty allowed it to notably reduce cases of NT for *in vitro* models (94% overall) compared to state-of-the-art methods. For DA to *in vivo* settings, TUGDA improved over previous methods for patient-derived xenografts (9 out of 14 drugs) as well as patient datasets (significant associations in 9 out of 22 drugs). TUGDA’s ability to avoid NT thus provides a key capability as we try to integrate diverse drug-response datasets to build consistent predictive models with *in vivo* utility.

**Availabilityand implementation:**

https://github.com/CSB5/TUGDA.

**Supplementary information:**

Supplementary data are available at *Bioinformatics* online.

## 1 Introduction

Advances in DNA sequencing technologies have galvanized a paradigm shift in medicine from a one-size-fits-all approach to precision medicine, that is tailored to stratified populations based on molecular information (Chae *et al.*, 2017). In oncology, an appreciation of the molecular diversity of cancers and limitations of standard-of-care treatments have further driven this interest toward patient-specific options based on re-purposing drugs and identifying targeted drug combinations (Brown and Elenitoba-Johnson, 2020). The availability of a large number of cancer cell lines has provided ready models for collecting drug response data (Iorio *et al.*, 2016). In combination with detailed omics profiles, these datasets present a unique opportunity to advance precision oncology based on state-of-the-art machine learning techniques (Jiang *et al.*, 2018).

The complexity inherent in biological systems and omics data poses two main challenges in learning models that could have clinical utility. Firstly, the high-dimensionality of omics data relative to the number of data points available can impact the generalizability of the models that are learnt (Azuaje, 2016). Joint models that predict response for many drugs in a multi-task learning (MTL) setting have been widely used to alleviate this limitation (Costello *et al.*, 2014; Suphavilai *et al.*, 2018; Wang *et al.*, 2017; Zhang and Yang, 2018). Secondly, while cell line datasets are typically used to learn predictive models, they are not expected to capture key aspects relevant to *in vivo* response including tumor heterogeneity and microenvironment, immune response and overall patient health (van Staveren *et al.*, 2009). Previous works (Geeleher *et al.*, 2014, 2017; Sakellaropoulos *et al.*, 2019) assumed that batch effects were the main origin of differences to correct for between models, without directly addressing biological variations. Recently, some methods have sought to use domain adaptation (DA) techniques to bridge the *in vitro* to *in vivo* gap (Mourragui *et al.*, 2019, 2020; Sharifi-Noghabi *et al.*, 2020).

An underlying principle shared for MTL and DA techniques is that *transfer learning*, whether it is across tasks or domains, needs generalization of information through shared representations. Inability to do this effectively leads to negative transfer (NT) where predictive performance for target tasks or domains is instead hampered relative to single-task learning (STL) (Zhang *et al.*, 2020). For MTL, this can happen when unrelated tasks are learnt together (potentially addressed by quantifying *task relatedness* as in GO-MTL, Kumar and Daumé, 2012) or when poor predictors adversely impact the shared representation (potentially addressed by weighting transfer flows based on task loss as in AMTL, Lee *et al.*, 2016 and its extension Deep-AMTFL, Lee *et al.*, 2018). For DA, NT can occur when there is weak or no similarity between domains (Kouw and Loog, 2021) and the method PRECISE (Mourragui *et al.*, 2019) seeks to address this for drug response prediction via a robust manifold alignment process. A refinement of this idea, TRANSACT (Mourragui *et al.*, 2020), uses Kernel-PCA based sub-space alignment to further capture non-linear relationships between samples from *in vitro* and *in vivo* domains. However, to learn the similarity between domains, existing DA methods either do not take into account the conditional distributions ($P_{s}(Y|X)$ and $P_{t}(Y|X)$ for drug response *Y* given gene expression *X* in source *s* and target *t*), obtaining a subset of shared features that might be unrelated to drug response (Mourragui *et al.*, 2019, 2020), or rely on the covariate-shift assumption (Sharifi-Noghabi *et al.*, 2020), where marginal distributions for features ($P_{s}(X)$ and $P_{t}(X)$, for tasks/domains *s* and *t*) are allowed to vary while the conditional distribution for drug response is assumed to be the same ($P_{s}(Y|X)=P_{t}(Y|X)$) (Kouw and Loog, 2021; Zhao *et al.*, 2019). This assumption can often lead to NT (Rampášek, 2020; Zhao *et al.*, 2019) when e.g. drugs that are effective *in vitro* do not successfully translate to the clinical setting (Wilding and Bodmer, 2014).

We present a unified transfer learning approach (TUGDA) for MTL and DA that leverages task/domain uncertainty (rather than loss) and a relaxed covariate-shift assumption to improve robustness of drug response prediction. Specifically, TUGDA captures both *aleatoric* (Kendall and Gal, 2017) and *epistemic* (Kendall *et al.*, 2018) uncertainties, and uses them to weight the task/domain to feature transfer. In addition, TUGDA relaxes the covariate-shift assumption across domains ($P_{s}(Y|X)\approx P_{t}(Y|X)$) for tasks with low confidence predictions using shared domain features. Our evaluations against state-of-the-art methods show that the use of uncertainties in guiding task-to-feature transfer reduces cases of negative transfer 94% overall and by 50% in harder cases that have limited *in vitro* data. For *in vivo* settings, TUGDA outperformed previous methods in transferring drug response predictions to both patient-derived xenograft (PDX) and patient tumors. Overall, TUGDA represents a novel unified framework to leverage information from *in vitro* and *in vivo* settings, and robustly predict cancer drug responses from molecular profiles.

## 2 Materials and methods

### 2.1 Definitions and preliminaries

We define a dataset $C={\left\{X_{t},y_{t}\right\}}_{i=1}^{T}$ consisting of $X_{t}\in R^{N_{t}\times d}$ gene expression profiles (*d* genes) and $y_{t}\in R^{N_{t}\times 1}$ drug response values for *T* different drugs and *N_t_* different data points (cell lines, xenografts or patients). In an MTL setting, we jointly learn predictive models for all *T* tasks under the following general framework:

(1) $$\underset{W}{\min}\sum_{t=1}^{T}\ell \left(w_{t};X_{t},y_{t}\right)+R(W),$$

where ℓ is the loss function (e.g. mean squared error, in our case) applied to each task *t*, with $w_{t}$ representing task-specific parameters as columns of $W\in R^{d\times T}$. The regularization term R is introduced to enforce priors over the task parameters and to improve generalization. This approach constrains joint learning in a naive manner (through the regularization term) and an approach to improve this is to assume that there exist shared latent bases across tasks (Argyriou *et al.*, 2008; Kumar and Daumé, 2012). We can represent this assumption and improve Eq. (1) as follows:

(2) $$\underset{L,S}{\min}\sum_{t=1}^{T}\left\{\ell \left(Ls_{t};X_{t},y_{t}\right)+\mu {\left\|s_{t}\right\|}_{1}\right\}+\lambda ||L|{|}_{F}^{2},$$

where ***W*** from Eq. (1) is decomposed as W=LS, with $L\in R^{d\times k}$ representing the set of *k* latent bases, and $S\in R^{k\times T}$ is the matrix containing vectors $s_{t}$ to combine those bases. The R term from eq. (1) is then replaced to constrain ***L*** to be $\ell_{2}$ regularized while $s_{t}$ needs to be $\ell_{1}$ sparse, with the hyperparameters *μ* and *λ* controlling the extent of regularization. This framework can be extended to take advantage of neural networks and use multiple layers of shared features followed by a task-specific layer. Here we assume that ***L*** and ***S*** are parameters for the first and the second (task-specific) hidden layers, respectively. The approach in Eq. (2) tries to reduce the risk of negative transfer by forcing unrelated tasks to use disjoint latent spaces. Nevertheless shared bases are trained without consideration of the quality of task-predictors, allowing for noisy and unreliable predictors to be the source of NT (Lee *et al.*, 2018). Assuming that task loss is a proxy for task reliability, the transfer from task-to-features can be guided (Lee *et al.*, 2016, 2018) by extending Eq. (2) as follows:

(3) $$\begin{array}{c} \overset{}{\underset{L,S,A}{\min}}\sum_{t=1}^{T}\left\{\left(1+\alpha {\left\|a_{t}^{o}\right\|}_{1}\right)\ell \left(Ls_{t};X_{t},y_{t}\right)+\mu {\left\|s_{t}\right\|}_{1}\right\}\\ +\gamma ||Z-\Omega (\mathrm{ZSA})|{|}_{F}^{2}+\lambda ||L|{|}_{F}^{2}, \end{array}$$

where $Z=\Omega (X_{t}L)$ is the output of the first neural layer ***L*** followed by a non-linear activation function Ω [ReLU (Nair and Hinton, 2010) in our case], ***Z*** is interpreted as the shared features space and it is used by ***S*** (task-specific parameters) to predict drug responses. ***A*** is a matrix which controls the amount of transfer from task *t* to *k* features by the row vector $a_{t}^{o}$ (A’s row vector). An auto-encoder regularization is then imposed aiming to reconstruct the latent features ***Z*** with the model output Ω(ZSA). This feedback loop between ***Z*** and ***A*** imposed by the autoencoder is expected to control the influence of unreliable tasks (based on task-loss) into the shared feature space. The hyperparameter *α* is multiplied by the training loss ℓ to control the sparsity of $a_{t}^{o}$, thus breaking the symmetry of transfer to features by forcing transfer from high loss tasks to be more sparse. Despite this sophisticated formulation, the assumption that task loss is a proxy for reliability may be misleading, especially in cases of overfitting from limited *in vitro* training data (Hawkins, 2004).

### 2.2 Leveraging task uncertainty for multi-task learning

We aim to estimate two types of task uncertainties and explore their use as alternative weights for task-to-feature transfer (Kendall and Gal, 2017). The first type is *aleatoric* uncertainty which captures uncertainty due to inherent noise in the experimental data that is being modeled. Specifically, as shown by (Kendall *et al.*, 2018) *homoscedastic aleatoric* uncertainty in MTL settings captures the relative confidence between tasks. As this uncertainty does not vary with input data, we can interpret it as task uncertainty reflecting the amount of noise inherent in drug response measurements. Let $f^{w_{t}}(x)$ be the output function for input **x** and task-weight $w_{t}$, we have the following relationship for *aleatoric* uncertainty per task (*σ_t_*) in a regression setting:

(4) $$\ell_{\mathrm{aleatoric}}=\frac{1}{2\sigma_{t}^{2}}(\frac{1}{N_{t}}||y_{t}-f^{w_{t}}(x)|{|}^{2})+\text{log}\;\sigma_{t},$$

where *σ* is learnable along with model parameters. Intuitively from Eq. (4), *σ_t_* can been interpreted as loss attenuation when the model predictions are far away from ground truth. As prior work has shown that MTL is strongly impacted by relative weighting for task losses (Kendall and Gal, 2017), the use of *aleatoric* uncertainty in TUGDA could reduce NT by automatically learning optimal loss weights.

A second type of task uncertainty that is accounted for in TUGDA is *epistemic*, representing the uncertainty in model parameters (Kendall and Gal, 2017). To do so, TUGDA uses Bayesian neural networks (BNNs, where weights W∼N(0,I)) to quantify model prediction uncertainties (Goan and Fookes, 2020). We use dropout variational inference (Gal and Ghahramani, 2016) for approximate inference in our model during training and testing (Srivastava *et al.*, 2014), thus enabling sampling from an approximate posterior distribution for weights ($q_{\theta }^{*}(W)$, in a tractable family) that minimizes the Kullback-Leibler divergence to the true model posterior (Gal and Ghahramani, 2016). We therefore extend eq. (4) as:

(5) $$\ell_{\mathrm{BNN}}=\frac{1}{2\sigma_{t}^{2}}(\frac{1}{N_{t}}||y_{t}-f^{\hat{W_{t}}}(x)|{|}^{2})+\text{log}\;\sigma_{t},$$

where ${\hat{W}}_{t}$ is sampled from the approximate distribution $q_{\theta }^{*}(W)$. In this setting, predictions are obtained by forwarding each sample ***x*** though the model for *P* passes, with weights sampled according to dropout inference.

In the TUGDA framework, with BNN ***L*** and ***S***, and a decoder layer ***A*** to regularize the task-to-feature transfer, the *epistemic* uncertainty for a task *t* given a sample ***x*** is computed in *P* passes as:

(6) $$U_{t}\left(x\right)=\frac{1}{P}\sum_{p=1}^{P}{\left(S\left(L\left(x\right)\right)-\frac{1}{P}\sum_{p^{\prime }=1}^{P}S\left(L\left(x\right)\right)\right)}^{2}.$$

**Fig. 1.**
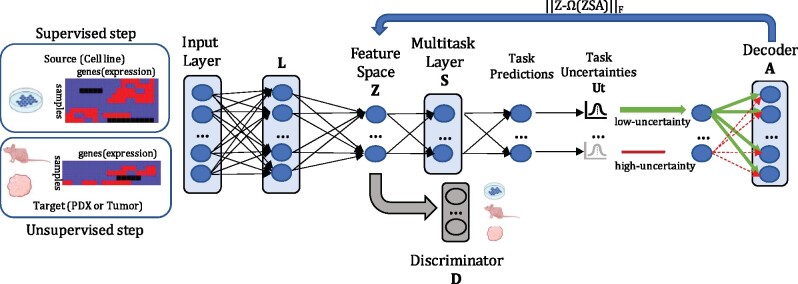
TUGDA framework for multi-task learning and domain adaptation in cancer drug response prediction. The layer L receives input data from cell lines (source data) in the supervised step or from other domains (PDX or patients, target data) in the unsupervised step and maps them to a latent space Z. Then, in the supervised step, the multi-task layer S uses these latent features to make predictions, as well as compute task-uncertainties $U_{t}$ for regularizing the amount of transfer from tasks/domains in A to the latent features in Z by employing an autoencoder regularization. Using adversarial learning, in both supervised and unsupervised steps, the discriminator D (in place to classify Z in different domains) receives the extracted features from Z and regularizes L to learn domain-invariant features. L, S, A and D consist of a single fully connected layer. Cell-line, PDX and tumor icons were Created with BioRender.com.

Following this, TUGDA’s novelty lies in formulating the use of task uncertainties to guide knowledge transfer from tasks *t* to features ***Z***, which is accomplished by extending Eq. (3) as follows:

(7) $$\begin{array}{c} \overset{}{\underset{L_{\theta },S_{\theta },A_{\theta }}{\min}}\ell_{\mathrm{MTLb}}=\sum_{t=1}^{T}\left\{\left(1+(U_{t}+{\left\|a_{t}^{o}\right\|}_{1}\right))\ell_{\mathrm{BNN}})+\mu {\left\|s_{t}\right\|}_{1}\right\}\\ +\gamma ||Z-\Omega (\mathrm{ZSA})|{|}_{F}^{2}+\lambda ||L|{|}_{F}^{2}, \end{array}$$

where $U_{t}$ is employed to weight $a_{t}^{o}$, thus forcing tasks with high-uncertainty to transfer less to the shared feature space ***Z*** (by the autoencoder regularization). A model representation for MTL with TUGDA is depicted in Figure 1 (blue layers) showing how the influence of unreliable tasks is attenuated by both *aleatoric* ($\ell_{\mathrm{BNN}}$) and *epistemic* ($U_{t}$) uncertainties, and how constraints for $a_{t}^{o}$ are learnt in an end-to-end fashion.

### 2.3 Domain adaptation with task uncertainty and relaxed covariate-shift assumption

To enable domain adaptation from *in vitro* to *in vivo* settings while avoiding NT for tasks where similarity between domains is limited, we extend eq. (7) by adding a Discriminator module *D* (Fig. 1, *D* in gray) that is responsible for classifying an extracted feature ***Z*** from *L*(*x*) into different domains (cell line, xenograft or patient tumor). The idea here is to use adversarial learning to match source (*in vitro*) and target (*in vivo*) marginal distributions (Ganin and Lempitsky, 2015). In this manner, we can describe the training process as a two-player game, where the module (L(x)) learns features that forces D(L(x))) toward confusion, while *D* needs to accurately classify domains (Fig. 1, blue and gray, done in both supervised and unsupervised steps). In the end, *L*(*x*) is expected to learn features *Z* that are domain-invariant and so we can describe the learning process as:

(8) $$\begin{array}{c} \underset{L_{\theta },S_{\theta }}{\min}\underset{D}{\max}\ell_{\mathrm{adv}}=-\frac{1}{n_{s}}\sum_{i=1}^{n_{s}}\left(\text{log}\;\left(D(L\left(x_{i}^{s}\right)\right)\right)\\ -\frac{1}{n_{t}}\sum_{i=1}^{n_{t}}\left(\text{log}\;\left(1-D(L\left(x_{i}^{t}\right)\right)\right) \end{array}$$

with *n_s_* and *n_t_* being the number of training samples from source (*in vitro*) and target (*in vivo*), respectively. To enable this adversarial training, we employed the Gradient Reversal Layer (GRL) approach (Ganin and Lempitsky, 2015) that works by flipping the sign of gradients that flow through *D* to the network during back-propagation. By adding a discriminator module *D* we end up with a framework that jointly learns a shared space between models (aligns the marginals) and uses these features to predict cancer drug response in an MTL setting. As we regularize transfer from task-to-features using task-uncertainties, we constrain our model (by the $a_{t}^{o}$ sparsity in *A*) to transfer less from predictions with high uncertainty based on shared features from different domains and tasks. An important by-product of this formulation is the relaxation of the covariate-shift assumption for transferring information from high-uncertainty predictors with the basis that they are less likely to retain predictions across domains. With this, TUGDA is trained in an end-to-end fashion as follows:

(9) $$\underset{L,Z,S,A}{\min}\underset{D}{\max}\ell_{\text{final}}=\ell_{\mathrm{MTLb}}+\lambda_{\text{adv}}\ell_{\text{adv}}$$

with $\lambda_{\text{adv}}$ as a hyperparameter which controls the influence of adversarial training.

## 3 Results

### 3.1 TUGDA reduces negative transfer in multi-task learning of *in vitro* drug responses

#### 3.1.1 Dataset and baselines

To evaluate the MTL performance of TUGDA (Fig. 1, blue), we used the *Genomics of Drug Sensitivity in Cancer* (GDSC) database (Iorio *et al.*, 2016) to obtain cell line drug response and transcriptomic data. Following the steps in Mourragui *et al.* (2020) to pre-process data, we obtained a matrix of normalized gene expression values for 806 cell-lines and 1780 genes (Hoogstraat *et al.*, 2014), in addition to response values for 200 drugs. As prior work has shown that regularized linear models often yield state-of-the-art results (Costello *et al.*, 2014; Jang *et al.*, 2014), we employed Ridge linear regression as the single-task learning baseline (we also experimented with Elastic Net as the baseline [median MSE = 2.78], but Ridge presented better overall performance [median MSE = 2.26], thus suited to explore NT cases). We then compared TUGDA with the state-of-the-art neural network-based multi-task learners GO-MTL (Kumar and Daumé, 2012), AMTL (Lee *et al.*, 2016) and Deep-AMTFL (Lee *et al.*, 2018), that are designed to avoid NT behavior. By combining the input layer and the module *L* with GO-MTL and AMTL we obtained two extended baselines that we refer to as Deep-GO-MTL and Deep-AMTL (Dizaji *et al.*, 2020; Lee *et al.*, 2016, 2018), respectively. Thus, all deep neural network models share the same number of layers until the prediction step (Input layer, L and S; Fig. 1), and the differences are only in terms of the regularization used. We performed 3-fold nested cross-validation (Varma and Simon, 2006) to report MTL performance. In this process, we select hyperparameters based on validation performance in the inner loop. The best performing model of the inner loop is evaluated on an outer test fold (unseen cell lines). This process obtains a performance estimate unbiased by hyperparameter selection. We searched for the best set of hyperparameters (list and range in Supplementary Note S2) using the *Tree-structured Parzen Estimator* algorithm (Bergstra *et al.*, 2011).

#### 3.1.2 Results with cell line data

**Fig. 2.**
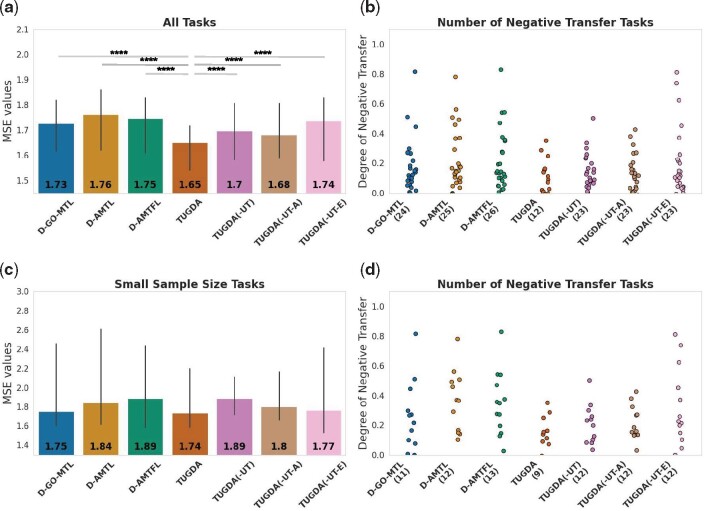
MTL performance evaluation using *in vitro* datasets. (**a**) Barplots (error bars represent standard deviation) showing MSE across tasks for different models including state-of-the-art methods (Deep-GO-MTL, Deep-AMTL, Deep-AMTFL), and TUGDA and its ablated variants (median MSE values are shown on the bottom along with statistical significance bars on top, where * stands for digits after the decimal p-value point i.e. ‘****’ signifies 1e-4), (**b**) Strip plots comparing the degree of negative transfer and the number of such tasks (shown in parenthesis). (**c**) and (**d**) Barplots and strip plots comparing MSE and NT for tasks with smaller sample sizes (19 tasks)

Models were trained to predict log IC50 values (concentration which kills 50% of cells; log-transformed) and compared in terms of mean squared error (MSE) distribution across all 200 drugs. As can be seen in Figure 2a (Supplementary Fig. S1a, full distribution), TUGDA improves over prior methods with the lowest median MSE of 1.65 and the highest Pearson correlation of 0.51 (Supplementary Fig. S1c). Higher performances were also observed in our ablation analysis, which consists of the following setup: TUGDA(-UT-E) is solely based on *aleatoric* uncertainty; TUGDA(-UT-A) uses *epistemic* uncertainty; and TUGDA(-UT) uses both uncertainty types but excludes the use of $U_{t}$ to weight $a_{t}^{o}$, i.e. feedback loop from *A* to *Z* will not take into account task-uncertainties. This analysis suggests that *epistemic* uncertainty plays an important role in TUGDA’s performance when compared to *aleatoric* uncertainty, but the full model is key in this dataset (Fig. 2a). We also employed Wilcoxon signed-rank test to compare TUGDA’s performance with the baselines and observed that TUGDA is significantly better than all baseline methods (Fig. 2a, significance bars and asterisks on top).

To quantify NT behavior, STL-based MSEs were subtracted from corresponding MTL-based MSEs s.t. positive values indicate NT (Supplementary Fig. S2, full distribution). As shown in Figure 2b, TUGDA presented the fewest number of NT cases (12 out of 200 tasks, 94% of tasks with no NT), reducing the number of tasks with NT by 50% relative to the next best method (Deep-GO-MTL). We next focused our analysis of performance on the more challenging tasks with smaller sample sizes (19 out of 200 drugs; where sample size median is 49 and maximum is 382) compared to the rest (sample size minimum is 716 and median is 745). We devised this experimental setup to reflect a more realistic scenario where drug response data can be limited. Here again, TUGDA improved over the existing methods Deep-AMTL and Deep-AMTFL in terms of median MSE (Fig. 2c), and the ablation analysis highlights the utility of the full model. As can be seen from Figure 2d, NT cases were clearly enriched in this set of 19 tasks and TUGDA reduces the number from 11 (for the next best method, Deep-GO-MTL) to 9 (52% of tasks without NT; reduction of NT tasks by 18% relative to Deep-GO-MTL). Taken together, these results highlight the importance of addressing task-uncertainty in MTL settings and TUGDA’s utility in more realistic pharmacogenomic settings for new drugs.

### 3.2 TUGDA provides a robust approach for domain adaptation from *in vitro* to *in vivo* response prediction

#### 3.2.1 Datasets and baselines

We evaluated the unified TUGDA framework (Fig. 1, blue and gray modules) against existing unsupervised DA methods for transferring cancer drug responses from cell lines (*in vitro*) to two different *in vivo* settings, patient-derived xenografts (PDX) and patient tumors. PDX data was obtained from the Novartis Institutes for Biomedical Research (Gao, 2015) containing gene expression profiles (*n* = 399) and drug responses values. Patient tumor gene expression profiles were obtained from TCGA (Network *et al.*, 2013) as well as curated response data from Ding *et al.* (2016). All cell line, PDX and tumor data were processed using the same pipelines, with pre-processing steps and experimental setup as proposed in Mourragui *et al.* (2020). As baselines for both PDX and patient tumor predictions we employed (extended from Mourragui *et al.*, 2020) the following: (i) an Elastic Net regression trained solely on cell line data. (ii) An Elastic Net regression trained solely on batch corrected cell line data (Elastic Net + Combat) approach similar to Geeleher *et al.* (2017). (iii) Deep Learning model (DL) (Mourragui *et al.*, 2020), (iv) Deep Learning + Combat (DL + Combat), similar to Sakellaropoulos *et al.* (2019), as well as the unsupervised DA approaches, (v) PRECISE (Mourragui *et al.*, 2019) and (vi) TRANSACT (Mourragui *et al.*, 2020) (see implementation details for all baselines in Supplementary Note S4).

Similar to previous UDA methods (Mourragui *et al.*, 2019, 2020), TUGDA is based on transductive learning (Kouw and Loog, 2021), where in the unsupervised learning step (Fig. 1) all target (ignoring labels) data is used to learn a domain-invariant space. The models are fine-tuned following the approach in Ganin and Lempitsky (2015), where the best set of hyperparameters was determined by minimizing MSE loss on source data (cell line AUC) using the domain-invariant features. This procedure was done for PDX data (list and range of hyperparameters in Supplementary Note S3, Supplementary Tables S2 and S3) and patient data (list and range of hyperparameters in Supplementary Note S3, Supplementary Tables S4 and S5). In both cases, the *Tree-structured Parzen Estimator* algorithm (Bergstra *et al.*, 2011) was used for searching hyperparameters.

#### 3.2.2 Results with PDX data

**Fig. 3.**
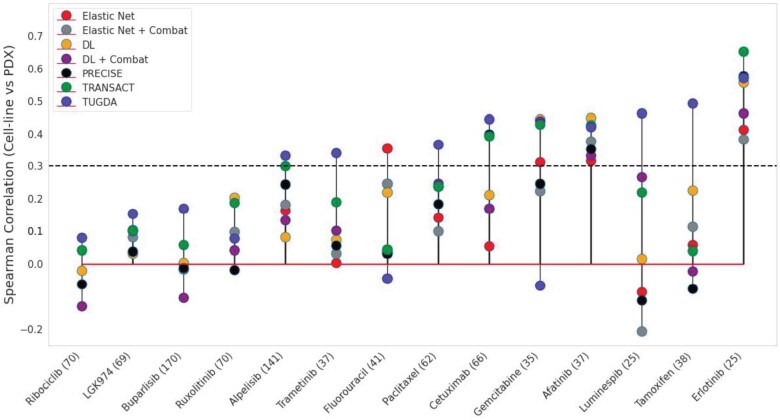
DA performance for predicting drug response in PDX models. Comparison of Spearman correlation between cell-line and PDX response values for 14 drugs across different models. Numbers in parenthesis represent PDX sample size. The dashed line stands for a threshold for moderate or higher correlation

We evaluated the transfer of drug responses from GDSC cell-lines to PDX data based on 14 shared drugs (extended seven drugs from Mourragui *et al.*, 2020) and computed Spearman correlations for predicted (AUC) and measured response values in the PDX setting (PDX best average response, lower values are related to sensitivity). Out of 14 drugs, TUGDA provided the highest Spearman correlation for 9 drugs (Fig. 3, Alpesilib, Buparlisib, Cetuximab, LGK974, Luminespib, Paclitaxel, Ribociclib, Tamoxifen and Trametinib), while DL, TRANSACT and Elastic Net were the best methods for three (Afatinib, Gemcitabine and Ruxolitinib), one (Erlotinib) and one (Fluorouracil) drugs, respectively. Furthermore, when examining these results for moderate or higher correlations, TUGDA presented 8 out 14 drugs above this threshold (0.3, dashed line Fig. 3), followed by TRANSACT and Elastic Net with 5 and 4 drugs, respectively. Investigating the learnt feature space, we observed that cell-lines and PDX samples from the same tissue tend to cluster together, showing that the model infers a biologically appropriate *in vitro* to *in vivo* transformation (See Supplementary Note S6, Supplementary Fig. S4).

#### 3.2.3 Results with patient tumor data

For patient tumor data, we evaluated performance for transferring drug response predictions from cell-lines to patients based on 22 drugs shared in GDSC and TCGA (extended 5 drugs from Mourragui *et al.*, 2020). As analyzed previously (Ding *et al.*, 2016; Mourragui *et al.*, 2020), TCGA drug responses were categorized into two groups, *Responders* (‘Complete Response’ and ‘Partial Response’) and *Non-responders* (‘Stable Disease’ and ‘Progressive Disease’). Despite several additional sources of variation in patient response data (tumor heterogeneity and environment, immune response, patient health status), TUGDA showed significant associations for nine drugs including Bleomycin, Carboplatin, Dacarbazine, Docetaxel, Doxorubicin, Paclitaxel, Premetexed, Tamoxifen and Vinblastine (Table 1; one-sided Mann-Whitney test between predicted AUC responses for Responders and Non-responders; *P*-value < 0.05), improving on TRANSACT (7 drugs), DL (7 Drugs), PRECISE (6 drugs), DL + Combat (4 drugs), Elastic Net + Combat (4 drugs) and Elastic Net (4 Drugs) baselines. Moreover, we compared against all baselines in terms of effect size (effect size associated with the Mann-Whitney test divided by the sample size) and noted that TUGDA outperformed them with 7 drugs presenting significant associations and the largest effect size (blue values, Table 1). In comparison, TRANSACT, DL, Elastic Net and Elastic Net + Combat, presented 5, 2, 1 and 1 drug, respectively, with this property. Collectively, out of 31 different drugs tested across the two domains, TUGDA captured significant associations (Spearman correlation from PDX and largest effect-size for significant drugs in TCGA data) for 14 drugs (Alpesilib, Buparlisib, Cetuximab, LGK974, Luminespib, Ribociclib, Tamoxifen, Trametinib from PDX and Bleomycin, Dacarbazine, Docetaxel, Doxorubicin, Pemetrexed, Vinblastine from TCGA data). In comparison the next best method, TRANSACT, captured significant associations for 6 out of 31 drugs (Erlotinib from PDX, and Carboplatin, Cisplatin, Gemcitabine, Paclitaxel and Trastuzumab from TCGA data). These results confirm TUGDA’s relative utility for transfer learning of drug responses from *in vitro* to *in vivo* models (TUGDA also presented the largest improvements relative to the next best method, see Supplementary Note S5). As was the case for the PDX model, the UMAP projection of the learnt feature space from TUGDA largely clusters cell-line and patient tumor data by tissue type (see Supplementary Note S6, Supplementary Fig. S5), highlighting that it can successfully learn shared biological properties.

**Table 1.** DA performance for predicting drug response in patient data

Drug	Samples	Elastic Net	Elastic Net+Combat	DL	DL+Combat	PRECISE	TRANSACT	TUGDA
Bicalutamide	17	0.142 [0.71]	0.116 [0.74]	0.285 [0.62]	0.525 [0.50]	0.116 [0.74]	0.244 [0.64]	0.330 [0.60]
Bleomycin	53	0.128 [0.65]	0.494 [0.50]	0.332 [0.56]	0.528 [0.49]	0.082 [0.68]	0.091 [0.67]	**0.043 [0.72]**
Carboplatin (Cisplatin)	166	0.262 [0.53]	0.333 [0.52]	0.114 [0.56]	0.428 [0.51]	**0.023 [0.59]**	**0.004 [0.63]**	**0.023 [0.59]**
Cetuximab	19	0.484 [0.51]	0.419 [0.53]	0.484 [0.51]	0.419 [0.53]	0.484 [0.51]	0.077 [0.70]	0.298 [0.58]
Cisplatin	308	**3.6e-4 [0.64]**	**4.6e-4 [0.63]**	**6.9e-5 [0.65]**	**8.5e-4 [0.60]**	**2.1e-5 [0.66]**	**7.2e-7 [0.69]**	0.244 [0.53]
Cyclophosphamide	102	0.491 [0.50]	0.402 [0.53]	0.755 [0.42]	0.874 [0.36]	0.112 [0.65]	0.587 [0.47]	0.531 [0.49]
Dacarbazine (AICAR)	30	0.278 [0.56]	0.225 [0.58]	0.368 [0.54]	0.178 [0.60]	0.384 [0.53]	0.692 [0.48]	**0.014 [0.73]**
Docetaxel	102	0.447 [0.51]	0.674 [0.47]	0.326 [0.53]	0.564 [0.49]	0.762 [0.46]	0.115 [0.57]	**0.001 [0.69]**
Doxorubicin	101	0.216 [0.55]	0.977 [0.38]	0.347 [0.52]	0.965 [0.39]	0.998 [0.32]	0.703 [0.47]	**1.1e-4 [0.72]**
Epirubicin	25	0.113 [0.73]	0.113 [0.73]	0.129 [0.71]	0.239 [0.64]	0.677 [0.42]	0.190 [0.67]	0.615 [0.45]
Etoposide	84	**0.002 [0.77]**	**0.003 [0.76]**	**0.002 [0.77]**	**0.005 [0.74]**	**0.007 [0.73]**	**0.026 [0.68]**	0.582 [0.48]
Fluorouracil	186	0.763 [0.47]	0.896 [0.44]	0.747 [0.47]	0.848 [0.45]	0.800 [0.46]	0.361 [0.52]	0.251 [0.53]
Gemcitabine	156	**0.004 [0.62]**	**0.013 [0.60]**	**0.024 [0.59]**	0.063 [0.57]	**0.040 [0.58]**	**0.003 [0.63]**	0.114 [0.56]
Irinotecan	25	0.826 [0.38]	0.717 [0.43]	0.263 [0.59]	0.630 [0.46]	0.536 [0.49]	0.464 [0.52]	0.737 [0.42]
Oxaliplatin	66	0.246 [0.55]	**0.001 [0.73]**	**0.029 [0.64]**	**0.005 [0.69]**	**0.027 [0.65]**	**0.035 [0.64]**	0.987 [0.33]
Paclitaxel	160	0.429 [0.51]	0.114 [0.56]	**0.005 [0.62]**	0.291 [0.53]	0.129 [0.56]	**0.004 [0.63]**	**0.010 [0.61]**
Pemetrexed	38	0.517 [0.50]	0.124 [0.61]	0.506 [0.50]	0.336 [0.54]	0.179 [0.59]	0.368 [0.53]	**0.018 [0.70]**
Tamoxifen	23	0.825 [0.38]	0.989 [0.21]	0.790 [0.40]	0.943 [0.30]	0.487 [0.51]	0.896 [0.34]	**0.024 [0.76]**
Temozolomide	96	0.153 [0.60]	0.238 [0.57]	0.260 [0.56]	0.500 [0.50]	0.587 [0.48]	0.182 [0.59]	0.618 [0.47]
Trastuzumab (Afatinib)	16	**0.024 [0.96]**	0.117 [0.79]	**0.048 [0.89]**	**0.034 [0.93]**	**0.024 [0.96]**	**0.016 [1.00]**	0.468 [0.54]
Vinblastine	16	0.336 [0.57]	0.298 [0.59]	0.664 [0.44]	0.263 [0.60]	0.500 [0.51]	0.584 [0.48]	**0.022 [0.81]**
Vinorelbine	30	0.403 [0.53]	0.053 [0.71]	**0.035 [0.73]**	0.053 [0.71]	0.163 [0.63]	0.384 [0.54]	0.671 [0.45]

*Note*: Drug names in parenthesis are corresponding matches from GDSC. We report *P*-values (in bold for *P* < 0.05) and the effect-size in brackets. Blue colored values indicate significant associations with the largest effect size for a drug.

### 3.3 Interpretability of TUGDA’s predictions is supported by known drug mechanisms

To explore the interpretability of TUGDA’s learnt feature space we computed the weights (attributions) of each gene using the Integrated Gradients (IG) method (Sundararajan *et al.*, 2017) for the PDX and TCGA test samples projected onto TUGDA’s shared feature space. IG computes the gradient of the model prediction output (AUC) relative to its input features (gene expression), where positive or negative weights are associated with increases (high expression, resistance) or decreases (low expression, sensitivity) of the AUC output, respectively. We then looked for enriched pathways present in TUGDA predictions using the computed ranking for each gene based on IG scores, and a pre-ranked gene set enrichment analysis (FDR correction at 25%, 1000 permutations and the gene sets MSigDB c2 and BioCarta) (Mourragui *et al.*, 2020; Subramanian *et al.*, 2005).

Based on the top-ranked gene set for each drug, we observed strong associations between TUGDA’s latent feature space attribution and known drug response mechanisms. For example, IG analysis with TUGDA’s model identified overexpression of the interleukin-6 (*IL-6*) signaling pathway as a significant marker of resistance to Tamoxifen (FDR = 0.059, Supplementary Fig. S6a). This is consistent with the known role of *IL-6* secretion by cancer-associated fibroblasts for tamoxifen resistance in luminal breast cancers (Sun *et al.*, 2014a,b). Similarly for Paclitaxel (another drug with a predictive TUGDA model for PDX [Fig. 3] and patient data [Table 1]), we noted enrichment of genes linked as potential microRNA 302 targets (FDR = 0.233, Supplementary Fig. S6b). The microRNA 302 family regulates cell proliferation and differentiation, and high expression of *miR-302* has been associated with Paclitaxel resistance (Greer Card *et al.*, 2008; Wu *et al.*, 2019). Among other associations, we noted MET signaling for Docetaxel (FDR = 0.202, Supplementary Fig. S6c) as observed in Kosaka *et al.* (2011), Wnt Signaling for Doxorubicin (FDR = 0.034, Supplementary Fig. S6d) which regulates resistance in breast cell lines (Martin-Orozco *et al.*, 2019), and activation of NFAT signaling for Trametinib (FDR = 0.061, Supplementary Fig. S6e) resistance as has been reported previously (Zhang *et al.*, 2017). Together, these observations support the notion that TUGDA’s framework captures relevant biological aspects of different drug response mechanisms which can be probed further using the IG method.

## 4 Discussion and conclusion

TUGDA’s strength lies in the fact that it represents a novel unified transfer learning approach for multi-task learning and domain adaptation that leverages the concept of task/domain uncertainty. These attributes align it to the fundamental challenges found in building predictive models for precision oncology, including sample size limitations, lack of curated *in vivo* data and violations of the covariate-shift assumption when taking into account drug responses. Our experiments show that TUGDA can provide notable benefits in a multi-task setting to reduce negative transfer, particularly when training data is limited. In addition, it shows promise as a way to robustly transfer information from *in vitro* data to *in vivo* settings, based on confidence in task predictions. In particular, for domain adaptation with patient data, we observed that TUGDA performed well for drugs that were often distinct and complementary to those from previous STL DA methods, potentially due to its multi-task learning formulation finding an alternate optimum that minimizes the error for more drugs (Zhang and Yang, 2018). However, as a side effect of this, for a subset of drugs TUGDA was not able to present strong performance relative to STL DA methods (e.g Fluorouracil and Gemcitabine for PDX data and Cisplatin, Etoposide, Gemcitabine, Oxaliplatin and Trastuzumab for patient data). A possible future direction is to explore which tasks should be learned together and which tasks should be automatically downgraded to STL (Standley *et al.*, 2020; Lozano and Swirszcz, 2012).

There a several potential pitfalls in the use of deep learning methods for computational biology, including unstable predictions (Mourragui *et al.*, 2020), overfitting and interpretability. TUGDA’s design seeks to address these by providing a stable training and prediction process (see Supplementary Note S8), and employing Bayesian neural networks, L1 and L2 regularizations for feature and task-specific layers, dropouts and task-uncertainties for regularizing task-to-feature transfer (instead of attention weights, Nguyen *et al.*, 2020) to avoid overfitting. To address the interpretability gap (Gilpin *et al.*, 2019), we explored and presented predictions for drugs with different mechanisms of action and trained on different domains (PDX or patient) that could be explained by the target’s pathway.

TUGDA’s approach to relaxing the covariate-shift assumption is a natural by-product of MTL using low-uncertainty features in a adversarial domain adaptation framework. This is distinct from prior work (Adel *et al.*, 2017) that is based on learning the probability of label changes across source and target domains and using this to weight transfer. In a recent study, the concept of *label-shift* has also been highlighted as a source of NT (Tan *et al.*, 2020). Intrinsic differences in cancer cell lines and patient tumors (e.g. the enrichment of genomic alterations and *in vitro* selection of subpopulations) make this scenario a likely one for domain adaptation in precision oncology. We envisage that TUGDA’s framework can be extended to alleviate NT in the marginal distribution of drug responses as well, advancing the goal of realistic precision oncology models further.

## Funding

This work was supported by funding from the Agency for Science, Technology and Research (A*STAR), Singapore and an A*STAR grant IAF-PP [A18A9b0060].

*Conflict of Interest*: none declared.

## Supplementary Material

btab299_Supplementary_Data
