# Supplementary material for: TUGDA: task uncertainty guided domain adaptation for robust generalization of cancer drug response prediction from in vitro to in vivo settings
Source: Bioinformatics. 2021 Jul 12;37(Suppl 1):i76–83. doi: 10.1093/bioinformatics/btab299 (PMC8275325; doi:10.1093/bioinformatics/btab299)
Supplement: btab299_Supplementary_Data [file btab299_supplementary_data.pdf]

Supplementary material for TUGDA: Task uncertainty guided domain adaptation for robust generalization of cancer drug response prediction from *in vitro* to *in vivo* settings

Rafael Peres da Silva[1,2], Chayaporn Suphavilai[1] and Niranjan Nagarajan[1,2,3]

[1] School of Computing, National University of Singapore, Singapore rafael@comp.nus.edu.sg
[2] Genome Institute of Singapore, A*STAR, Singapore suphavilai_chayaporn,nagarajann@gis.a-star.edu.sg
[3] Yong Loo Lin School of Medicine, National University of Singapore, Singapore

Supp 1 Multi-task learning - Additional Results

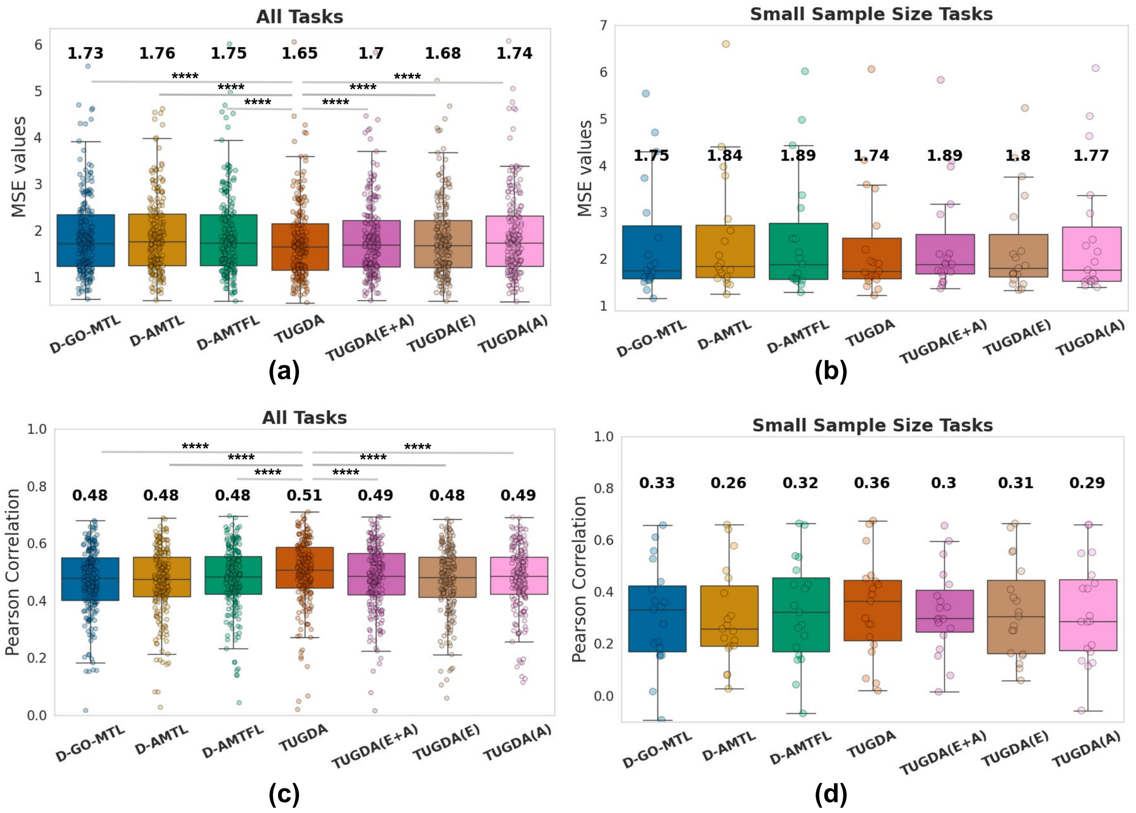

Supp Fig. 1. MTL performance evaluation using *in vitro* datasets.Boxplots showing MSE across all tasks (a) and for tasks with small sample size (b) for different state-of-the-art methods (Deep-GO-MTL, Deep-AMTL, Deep-AMTFL), and TUGDA and its ablated variants (median MSE or Pearson Correlation values are shown on top). Overlaid strip plots show individual data points. Corresponding plots for Pearson correlation are shown in (c) and (d). We observed that TUGDA's performance is significantly higher than all baseline methods (Wilcoxon signed-rank test; significance bars and asterisks on top).

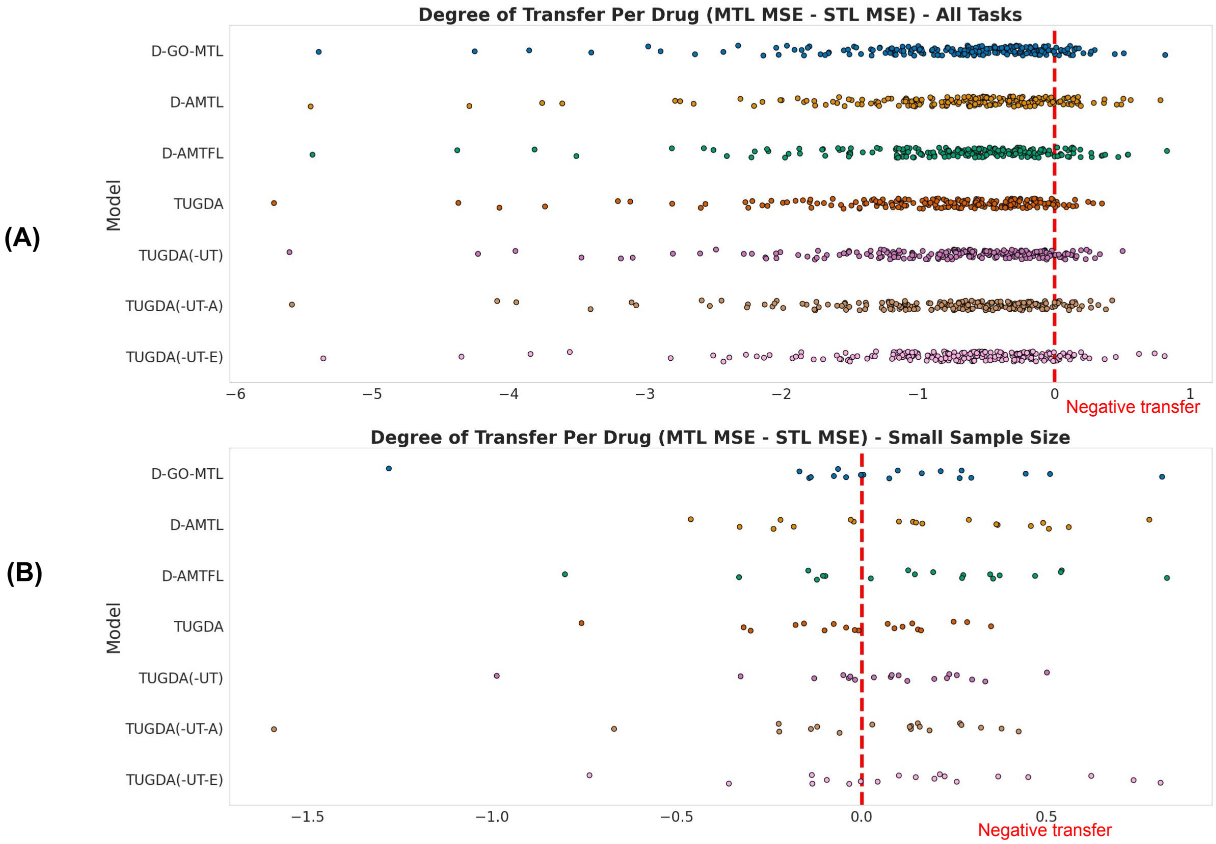

Supp Fig. 2. Degree of Transfer - Distribution. Strip plots showing the degree of transfer (MTL MSE - STL MSE) for each task per model. Data points at the right (left) of the red line show negative (positive) transfer. Distribution for (a) all tasks and (b) small sample size tasks.

Supp 2 Multi-task learning hyperparameters

We searched for the best set of hyperparameters for each MTL model in the following space detailed in Supp Table 1:

Supp Table. 1. Set of hyperparameters that were searched over in MTL experiments

Hyperparameters	Values
L Layer Units	64, 128, 256, 512, 1024
Z Layer Units	200, 300, 400, 500, 600, 700
Learning Rate (LR)	1e-2, 1e-3, 1e-4, 1e-5, 5e-2, 5e-3, 5e-4, 5e-5
Mu	1, 0.1, 0.01, 0.001, 0.0001
Lambda	1, 0.1, 0.01, 0.001, 0.0001
Gamma	1, 0.1, 0.01, 0.001, 0.0001
Alpha	1, 0.1, 0.01, 0.001, 0.0001
Dropout	0.1, 0.2, 0.3, 0.4, 0.5

For the ridge linear regression (STL model) we searched the α parameter for each drug using the following range: [1.e-05, 1.e-04, 1.e-03, 1.e-02, 1.e-01, 1.e+00, 1.e+01, 1.e+02, 1.e+03, 1.e+04, 1.e+05, 1.e+06, 1.e+07, 1.e+08, 1.e+09, 1.e+10]. We selected the best performing model based on the validation mean square error using the *Tree-structured Parzen Estimator* algorithm [2]. All TUGDA MTL models were implemented using Pytorch[6], Pytorch Lightning[3] and Optuna[1] for optimization.

Supp 3 Domain adaptation hyperparameters

For the domain adaptation experiments, we extended the search to include the following hyperparameters: 1) number of units in the layers of the domain discriminator D, 2) lambda disc (the hyperparameter controlling the adversarial learning influence) and 3) the discriminator batch size. As we are handling additional datasets, we extended the search space to increase the model's capacity. For experiments on domain adaptation from GDSC to PDX data the range is described in Supp Table 2 and the best settings can be found in Supp Table 3, and for GDSC to TCGA data the range is described in Supp Table 4 and the best settings can be found in Supp Table 5.

All TUGDA DA models were implemented using Pytorch[6], Pytorch Lightning[3] and Optuna[1] for optimization.

Supp 4 Domain Adaptation Baselines

We compared TUGDA against six baselines. For Elastic Net and Elastic Net (+Combat), we tunned α and L1 ratio hyperparameters by performing a grid-search 5-fold cross-validation on cell lines [5]. Deep Learning (DL) and Deep Learning + Combat (DL + Combat) [8] were reproduced following[8], where we randomly selected 30 sets of model parameters from the grid search and perform 5-fold cross-validation on cell lines as described in[8]. Following this, we then retrain the network on the complete GDSC dataset (using the best set of hyperparameters per drug). For the unsupervised domain adaptation methods PRECISE and TRANSACT, results were reproduced following[5] using the available source code (`https://github.com/NKI-CCB/TRANSACT_manuscript`). For PDX

Supp Table. 2. Set of hyperparameters searched in DA (PDX) experiments

Hyperparameters	Values
L Layer Units	512, 1024, 1500
Z Layer Units	800, 900, 1000
Learning Rate (LR)	1e-2, 1e-3, 1e-4, 1e-5, 5e-2, 5e-3, 5e-4, 5e-5
Mu	1, 0.1, 0.01, 0.001, 0.0001
Lambda	1, 0.1, 0.01, 0.001, 0.0001
Gamma	1, 0.1, 0.01, 0.001, 0.0001
Dropout	0.1, 0.2, 0.3, 0.4, 0.5
Lambda disc	0.1, 0.2, 0.3, 0.4, 0.5, 0.6, 0.7, 0.8, 0.9, 1.0
Batch size disc	32, 64, 128, 256, 300
Epochs	20, 30, 40, 50
Discriminator units	400, 500, 600, 700

Supp Table. 3. Best settings found for DA (PDX) with source MSE (cell line data)

Model	Set
TUGDA-DA-PDX	L layer Units: 1500, S and A layer Units: 800, LR: 0.001, Mu: 1, Lambda: 1, Gamma: 0.01, Dropout: 0.1, Discriminator units: 500, Epochs: 50, Batch size: 64, Lambda disc: 0.3

Supp Table. 4. Set of hyperparameters searched in DA (TCGA) experiments

Hyperparameters	Values
L Layer Units	1024, 1250, 1500
Z Layer Units	800, 900, 1000
Learning Rate (LR)	1e-2, 1e-3, 1e-4, 1e-5, 5e-2, 5e-3, 5e-4, 5e-5
Mu	1, 0.1, 0.01, 0.001, 0.0001
Lambda	1, 0.1, 0.01, 0.001, 0.0001
Gamma	1, 0.1, 0.01, 0.001, 0.0001
Dropout	0.1, 0.2, 0.3, 0.4, 0.5
Lambda disc	0.1, 0.2, 0.3, 0.4, 0.5, 0.6, 0.7, 0.8, 0.9, 1.0
Batch size disc	512, 1024
Epochs	20, 30, 40, 50
Discriminator units	500, 600, 700

Supp Table. 5. Best settings found for DA (TCGA) with source MSE (cell line data)

Model	Set
TUGDA-DA-TCGA	L Layer units: 1024, S Layer units: 900, LR: 0.001, dropout: 0.1, Mu: 0.01, Lambda: 0.0001, Gamma: 0.01, Disc layer units': 500, Epochs: 50, Batch size disc: 512, Lambda disc: 0.2

data, we used the linear kernel for PRECISE, while for TRANSACT, we studied the predictive performance for seven different γ (amount of non-linearity) values ranging from a set 1×10^{-5} to 1×10^{-2}. In this case we report test values for the best performer. For patient data (TCGA), we used a linear kernel for PRECISE and we used $\gamma = 5 \times 10^{-4}$ as recommended for TRANSACT.

All previous baselines were built with the help of Scikit-learn[7], Scipy[10], Numpy[4] and Pandas[9]

Supp 5 Domain Adaptation - Additional Results

In this section, we present bar plots showing improvements over the next best method (performance gain) on domain adaptation settings for PDX data (Supp Figure 3a), patient data (Supp Figure 3b) and both (Supp Figure 3c).

Supp 6 Structure retained in TUGDA's feature space

Here we present the UMAP projection of the feature space learnt by TUGDA. We observed that cell-line and PDX samples from the same tissue tend to cluster together for PDX (Supp Figure 4) and TCGA (Supp Figure 5) data. Similar to the previous result [5], less separation were observed between some tissue types.

Supp 7 Gene set enrichment analysis

Here we present the enrichment plots for the most significantly enriched pathway per drug, identified based on TUGDA's ranked integrated gradients scores using GSEA (Supp Figure 6).

Supp 8 Impact of initialization on deep learning results

As described in Mourragui et al 2020[5], the methods Deep Learning (DL) and Deep Learning + Combat (DL + Combat) [8] are prone to variations in results with different model initializations. Here we selected the best models (based on TCGA settings, Supp Table 5) and retrained them using 50 different initializations. As can be seen in Supp Figure 4a and b, TUGDA is more stable in terms of p-values when compared to the two previous Deep Learning methods [5,8]. We also performed Bartlett's test for equal variances and observed a larger number of significant associations when TUGDA's results presented less variance (Supp Figure 7a and b, top plot), highlighting that TUGDA is more stable than previous deep learning methods. Furthermore, Mourragui et al [5] found no correlation between performance on cell line data (GDSC) and performance on target data (TCGA) in DL methods [8], making it difficult to pick a model solely based on the source performance. Following this, as TUGDA is based on an MTL objective that aims to minimize error on all drugs at once, we inspected how the average source loss is related to the average performance on the target domain (TCGA data). To do so, for each set of hyperparameters tested for TUGDA-TCGA (Supp Table 4), we computed the correlation between the MSE on GDSC data and the effect size on TCGA data (Supp Figure 8a), as well as MSE on GDSC data and the number of significant drugs with TCGA data (Supp Figure 8b). A moderate (Supp Figure 8a) to strong correlation (Supp Figure 8b) suggest that TUGDA's tuning process is robust and one can rely on minimizing source error within TUGDA's framework to obtain better performance on target domain.

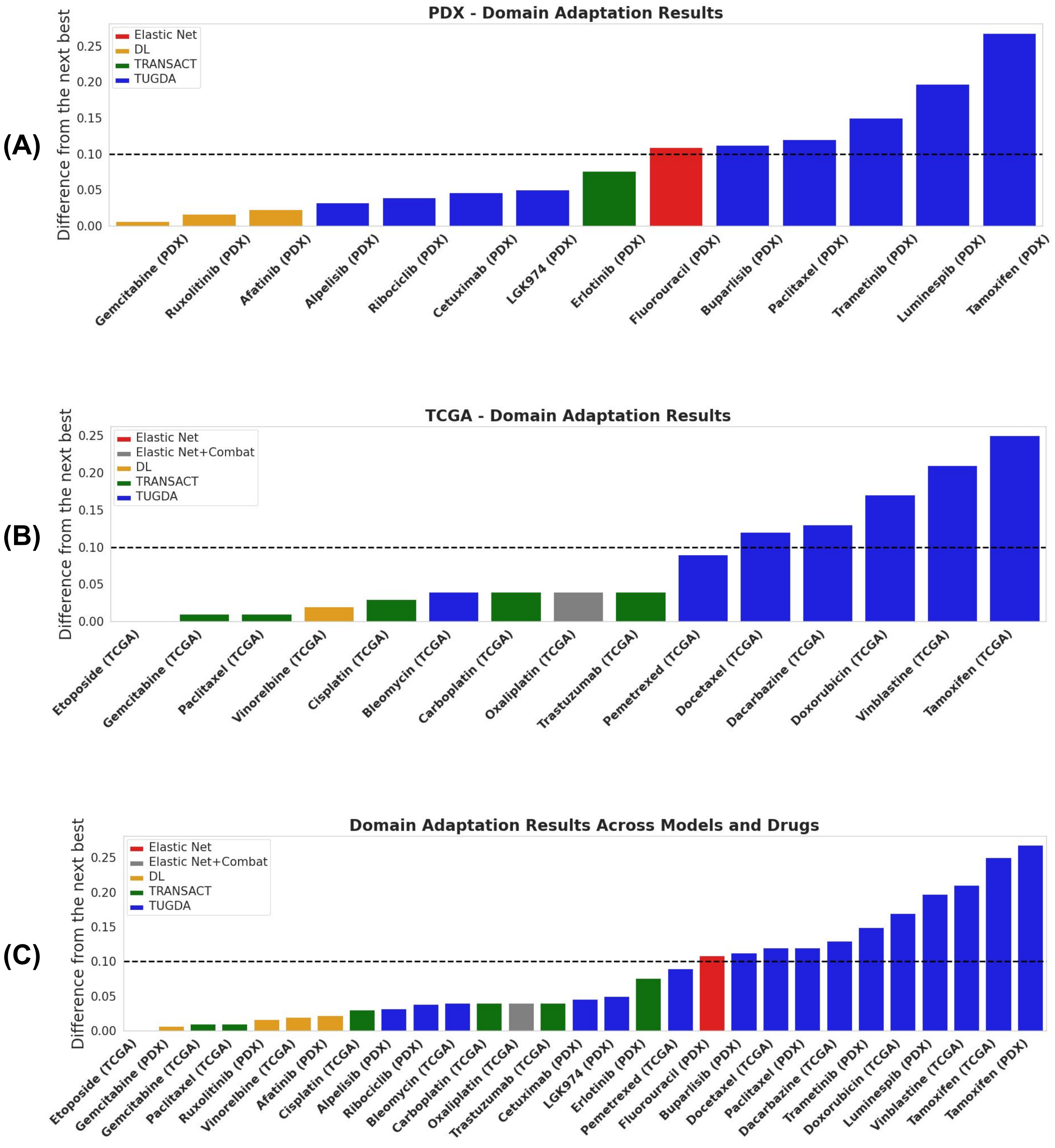

Supp Fig. 3. Performance gain results. Bar plots present the difference between the best performer (based on Spearman correlation or effect size for significant associations) per drug and domain and the next best method (performance gain) (a) with PDX data, (b) with TCGA data (for drugs with at least one model with significant results i.e. $p<0.05$), and (c) combining both domains. In all settings, TUGDA was observed to improve over other methods in providing the largest performance gains.

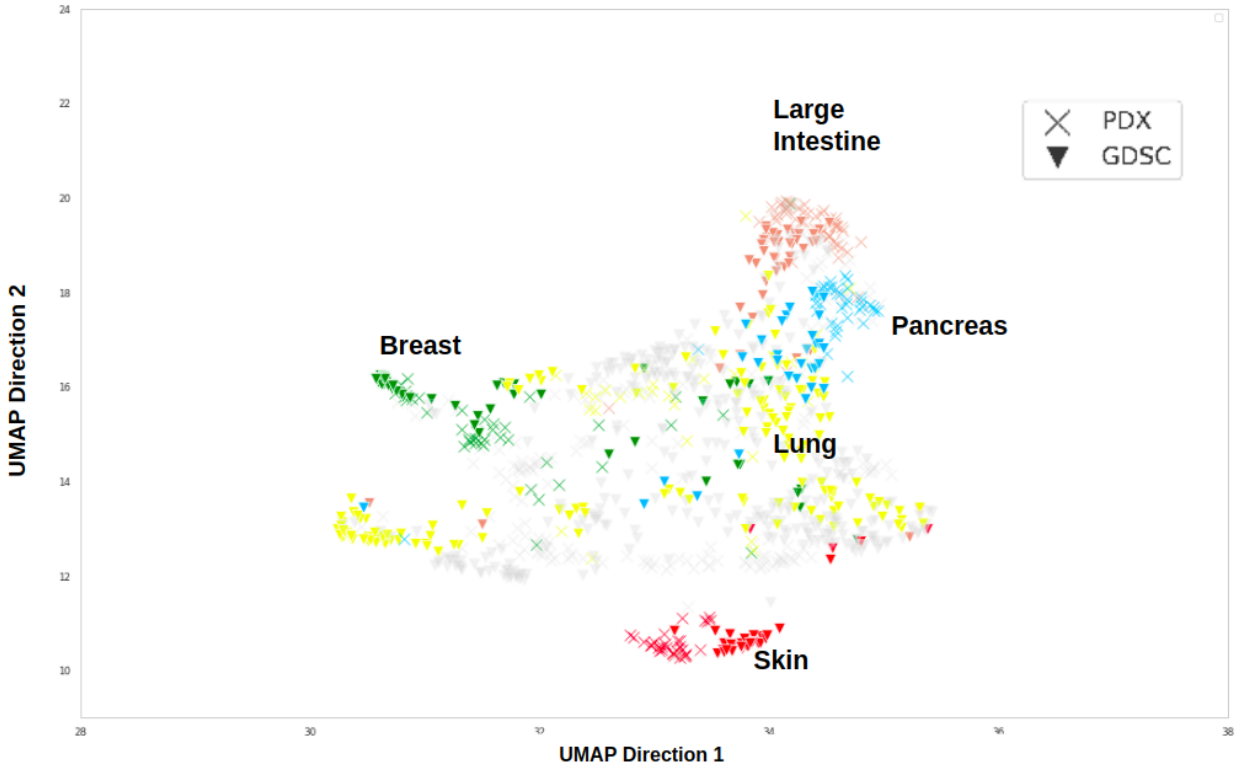

Supp Fig. 4. UMAP plot of feature space from TUGDA shows tissue-type specific clusters for cell-line and PDX data.

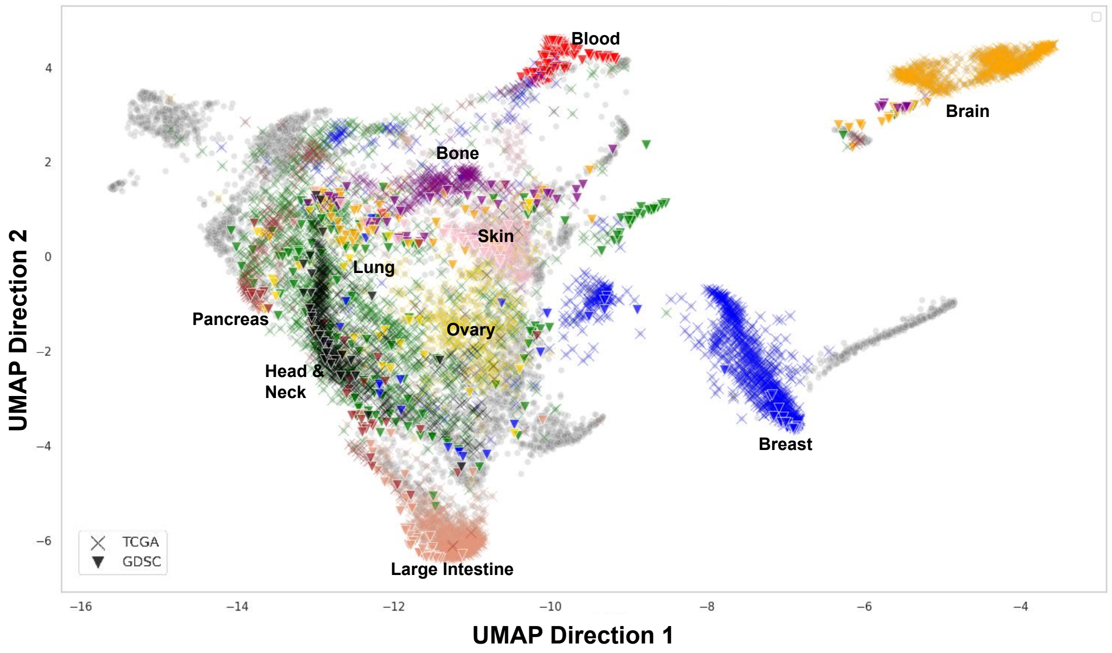

Supp Fig. 5. UMAP plot of feature space from TUGDA shows tissue-type specific clusters of cell-line and patient tumor data.

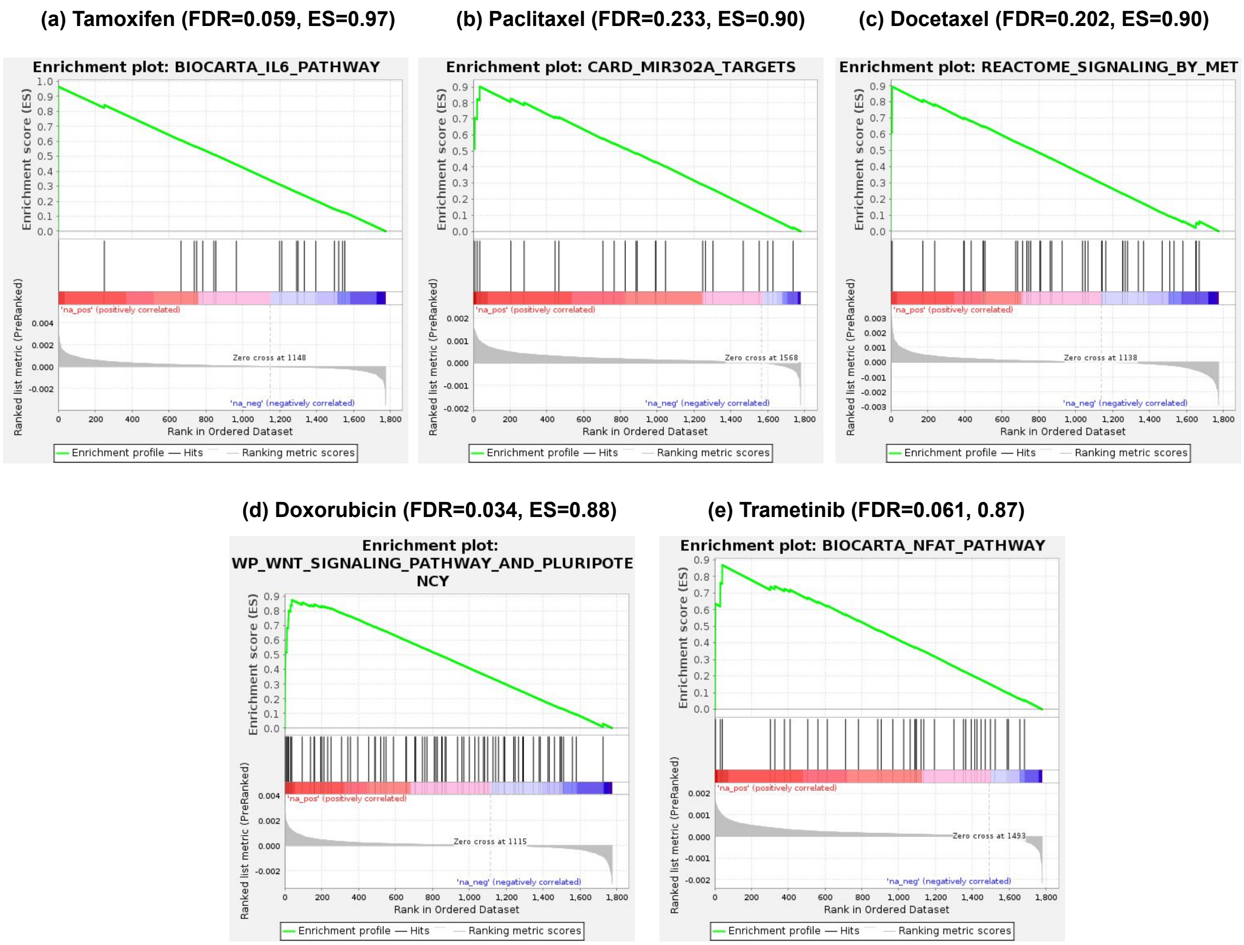

Supp Fig. 6. Gene Set Enrichment Analysis: the red to blue scale shows ranked gene weights (by integrated gradients scores), where red (blue) represents positive (negative) weights, contributing to higher (lower) AUC and thus drug resistance (sensitivity) predictions.

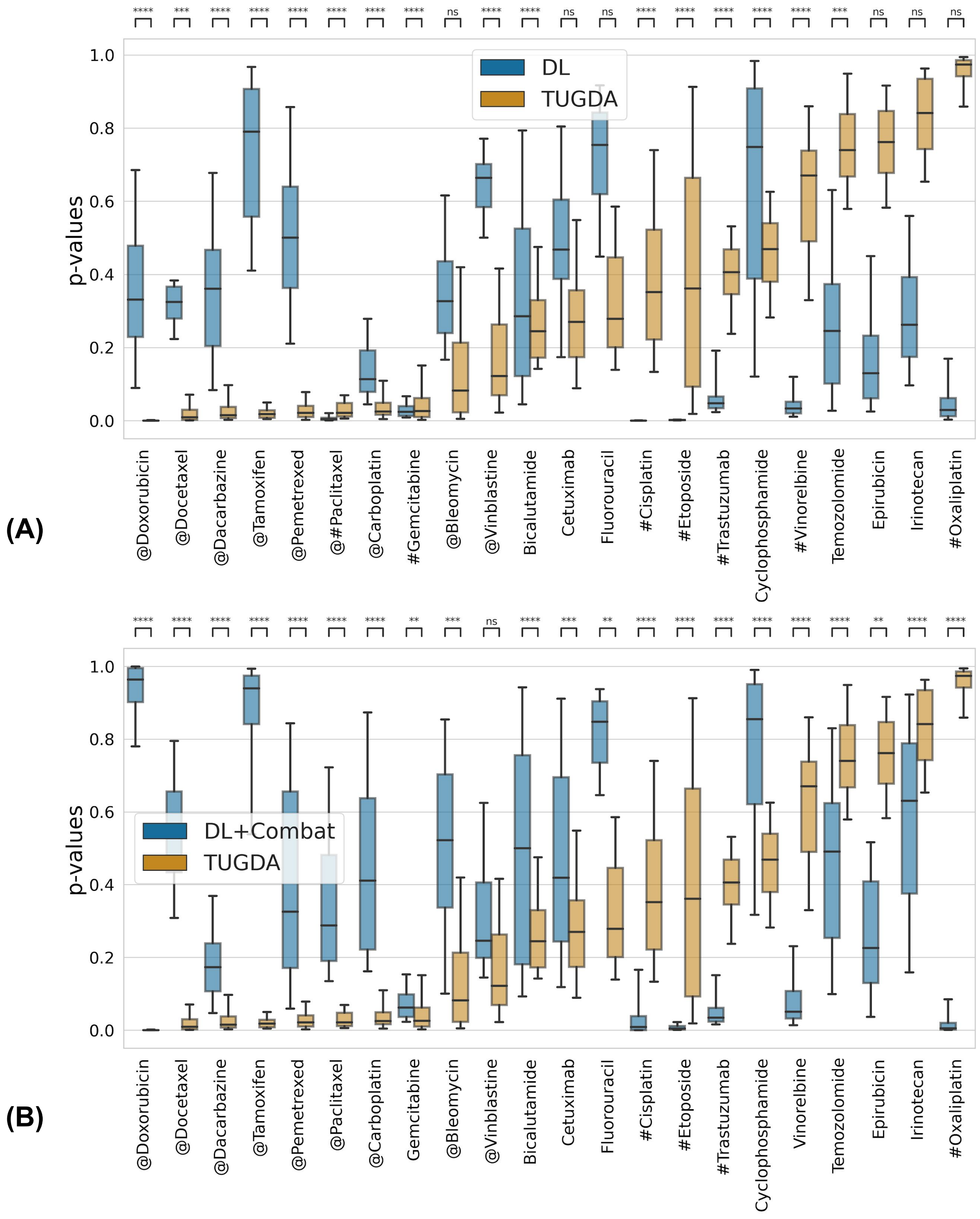

Supp Fig. 7. Comparison of TUGDA performance based on 50 different initializations against single-drug models for the TCGA dataset. @ indicates drugs for which TUGDA predicted significantly different response values between sensitive and resistant patients. # indicates drugs with significant values reported for DL (or + Combat version). On top of each plot we report the significance from Bartlett's test for homogeneity of variances. (a) TUGDA compared to DL (b) TUGDA compared to DL + Combat.

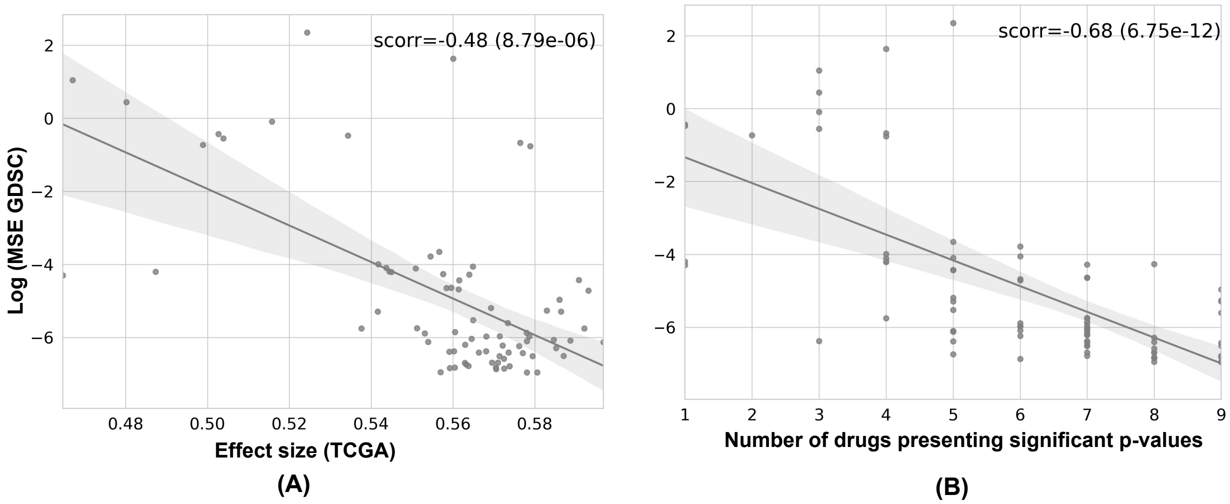

Supp Fig. 8. Predictive value of test loss on source data with TUGDA. Correlation between test loss and (a) effect size, (b) number of drugs presenting significant associations. In both evaluations we observed good correlation, suggesting that TUGDA's training optimization using cell-line labels is useful for improving prediction error in the patient domain.

References

1. Akiba, T., Sano, S., Yanase, T., Ohta, T., Koyama, M.: Optuna: A next-generation hyperparameter optimization framework. KDD '19, Association for Computing Machinery, New York, NY, USA (2019). https://doi.org/10.1145/3292500.3330701, https://doi.org/10.1145/3292500.3330701
2. Bergstra, J., Bardenet, R., Bengio, Y., Kégl, B.: Algorithms for hyper-parameter optimization. In: Proceedings of the 24th International Conference on Neural Information Processing Systems. p. 2546–2554. NIPS'11, Curran Associates Inc., Red Hook, NY, USA (2011)
3. Falcon, W.: Pytorch lightning. GitHub. Note: https://github.com/PyTorchLightning/pytorch-lightning **3** (2019)
4. Harris, C.R., Millman, K.J., van der Walt, S.J., Gommers, R., Virtanen, P., Cournapeau, D., Wieser, E., Taylor, J., Berg, S., Smith, N.J., Kern, R., Picus, M., Hoyer, S., van Kerkwijk, M.H., Brett, M., Haldane, A., Fernández del Río, J., Wiebe, M., Peterson, P., Gérard-Marchant, P., Sheppard, K., Reddy, T., Weckesser, W., Abbasi, H., Gohlke, C., Oliphant, T.E.: Array programming with NumPy. Nature **585**, 357–362 (2020). https://doi.org/10.1038/s41586-020-2649-2
5. Mourragui, S., Loog, M., Vis, D.J., Moore, K., Manjon, A.G., van de Wiel, M.A., Reinders, M.J., Wessels, L.F.: Precise+ predicts drug response in patients by non-linear subspace-based transfer from cell lines and pdx models. bioRxiv (2020). https://doi.org/10.1101/2020.06.29.177139, https://www.biorxiv.org/content/early/2020/07/28/2020.06.29.177139
6. Paszke, A., Gross, S., Massa, F., Lerer, A., Bradbury, J., Chanan, G., Killeen, T., Lin, Z., Gimelshein, N., Antiga, L., Desmaison, A., Kopf, A., Yang, E., DeVito, Z., Raison, M., Tejani, A., Chilamkurthy, S., Steiner, B., Fang, L., Bai, J., Chintala, S.: Pytorch: An imperative style, high-performance deep learning library. In: Wallach, H., Larochelle, H., Beygelzimer, A., d'Alché-Buc, F., Fox, E., Garnett, R. (eds.) Advances in Neural Information Processing Systems 32, pp. 8024–8035. Curran Associates, Inc. (2019), http://papers.neurips.cc/paper/9015-pytorch-an-imperative-style-high-performance-deep-learning-library.pdf
7. Pedregosa, F., Varoquaux, G., Gramfort, A., Michel, V., Thirion, B., Grisel, O., Blondel, M., Prettenhofer, P., Weiss, R., Dubourg, V., Vanderplas, J., Passos, A., Cournapeau, D., Brucher, M., Perrot, M., Duchesnay, E.: Scikit-learn: Machine learning in Python. Journal of Machine Learning Research **12**, 2825–2830 (2011)
8. Sakellaropoulos, T., Vougas, K., Narang, S., Koinis, F., Kotsinas, A., Polyzos, A., Moss, T.J., Piha-Paul, S., Zhou, H., Kardala, E., Damianidou, E., Alexopoulos, L.G., Aifantis, I., Townsend, P.A., Panayiotidis, M.I., Sfikakis, P., Bartek, J., Fitzgerald, R.C., Thanos, D., Mills Shaw, K.R., Petty, R., Tsirigos, A., Gorgoulis, V.G.: A deep learning framework for predicting response to therapy in cancer. Cell Reports **29**(11), 3367 – 3373.e4 (2019). https://doi.org/https://doi.org/10.1016/j.celrep.2019.11.017, http://www.sciencedirect.com/science/article/pii/S2211124719314883
9. pandas development team, T.: pandas-dev/pandas: Pandas (Feb 2020). https://doi.org/10.5281/zenodo.3509134, https://doi.org/10.5281/zenodo.3509134

10. Virtanen, P., Gommers, R., Oliphant, T.E., Haberland, M., Reddy, T., Cournapeau, D., Burovski, E., Peterson, P., Weckesser, W., Bright, J., van der Walt, S.J., Brett, M., Wilson, J., Millman, K.J., Mayorov, N., Nelson, A.R.J., Jones, E., Kern, R., Larson, E., Carey, C.J., Polat, İ., Feng, Y., Moore, E.W., VanderPlas, J., Laxalde, D., Perktold, J., Cimrman, R., Henriksen, I., Quintero, E.A., Harris, C.R., Archibald, A.M., Ribeiro, A.H., Pedregosa, F., van Mulbregt, P., SciPy 1.0 Contributors: SciPy 1.0: Fundamental Algorithms for Scientific Computing in Python. Nature Methods **17**, 261–272 (2020). https://doi.org/10.1038/s41592-019-0686-2
